# Extracellular vesicle DNA from human melanoma tissues contains cancer-specific mutations

**DOI:** 10.3389/fcell.2022.1028854

**Published:** 2022-12-01

**Authors:** Rossella Crescitelli, Stefan Filges, Nasibeh Karimi, Ornella Urzì, Tamara Alonso-Agudo, Anders Ståhlberg, Jan Lötvall, Cecilia Lässer, Roger Olofsson Bagge

**Affiliations:** ^1^ Sahlgrenska Center for Cancer Research and Wallenberg Centre for Molecular and Translational Medicine, Department of Surgery, Institute of Clinical Sciences, Sahlgrenska Academy, University of Gothenburg, Gothenburg, Sweden; ^2^ Sahlgrenska Center for Cancer Research and Wallenberg Centre for Molecular and Translational Medicine, Department of Laboratory Medicine, Institute of Biomedicine, Sahlgrenska Academy at University of Gothenburg, Gothenburg, Sweden; ^3^ Krefting Research Centre, Department of Internal Medicine and Clinical Nutrition, Institute of Medicine at Sahlgrenska Academy, University of Gothenburg, Gothenburg, Sweden; ^4^ Department of Biomedicine, Neurosciences and Advanced Diagnostics (Bi.N.D), University of Palermo, Gothenburg, Italy; ^5^ Department of Clinical Genetics and Genomics, Sahlgrenska University Hospital, Region Västra Götaland, Gothenburg, Sweden; ^6^ Department of Surgery, Sahlgrenska University Hospital, Region Västra Götaland, Gothenburg, Sweden

**Keywords:** tumor-derived extracellular vesicles, melanoma, DNA, ultrasensitive DNA sequencing, SiMSen-Seq

## Abstract

Liquid biopsies are promising tools for early diagnosis and residual disease monitoring in patients with cancer, and circulating tumor DNA isolated from plasma has been extensively studied as it has been shown to contain tumor-specific mutations. Extracellular vesicles (EVs) present in tumor tissues carry tumor-derived molecules such as proteins and nucleic acids, and thus EVs can potentially represent a source of cancer-specific DNA. Here we identified the presence of tumor-specific DNA mutations in EVs isolated from six human melanoma metastatic tissues and compared the results with tumor tissue DNA and plasma DNA. Tumor tissue EVs were isolated using enzymatic treatment followed by ultracentrifugation and iodixanol density cushion isolation. A panel of 34 melanoma-related genes was investigated using ultra-sensitive sequencing (SiMSen-seq). We detected mutations in six genes in the EVs (*BRAF*, *NRAS*, *CDKN2A*, *STK19*, *PPP6C*, and *RAC*), and at least one mutation was detected in all melanoma EV samples. Interestingly, the mutant allele frequency was higher in DNA isolated from tumor-derived EVs compared to total DNA extracted directly from plasma DNA, supporting the potential role of tumor EVs as future biomarkers in melanoma.

## 1 Introduction

Melanoma is the most aggressive skin cancer, and its etiology involves interactions between genetic susceptibility and environmental factors such as UV exposure (Rastrelli et al., 2014). Early diagnosis of melanoma is key for reducing mortality because melanoma cells can readily metastasize to different organs such as lymph nodes, lungs, liver, and brain (Hodi et al., 2010). Thanks to modern systemic treatment using immunotherapy and targeted therapy, the survival of melanoma patients has increased dramatically. However, the success of these therapies might be improved further using new biomarkers that will help in early detection (Hodi et al., 2016; Lim et al., 2018).

Tumor biopsies are used to acquire information about cancer type, risk factors, and genetic alterations, but they depend on the accessibility of the primary tumor or metastases. To overcome these limitations, liquid biopsies have emerged as a promising tool for cancer diagnosis and monitoring (Poulet et al., 2019; Lone et al., 2022).

Circulating tumor DNA (ctDNA) represents one of the most promising biomarkers for early cancer detection and disease monitoring (Campos-Carrillo et al., 2020; Salviano-Silva et al., 2022). DNA is normally contained in the nucleus of cells, but it can be released into the bloodstream upon cell death or EV release (Cheng et al., 2016). Several studies have demonstrated that ctDNA analysis can provide information about tumor stage (Diehl et al., 2005; Xu et al., 2017), tumor volume (Abbosh et al., 2017), and the presence of metastases (Chicard et al., 2018); and it is a helpful biomarker for melanoma staging (Knol et al., 2016; Long-Mira et al., 2018; Forschner et al., 2020). Nevertheless, the use of ctDNA has some disadvantages because it represents only a fraction of the total cell-free DNA (cfDNA), thus making the identification of low-frequency tumor mutations technically challenging. ctDNA also originates from dying cells, which might not represent the viable cells of the tumor (Jahr et al., 2001).

EVs are a heterogeneous group of bilayer membrane nanoparticles released by all cells, and seemingly even more so by tumor cells (van Niel et al., 2018; Xavier et al., 2020). Their cargo includes lipids (Dang et al., 2017), proteins (Doyle and Wang, 2019), and nucleic acids (Ridder et al., 2014), but this can vary depending on cell origin or the activity or phenotype of the cell. EVs can be isolated from many biological fluids, including blood (Xu et al., 2021), urine (Merchant et al., 2017), and saliva (Comfort et al., 2021). Moreover, EVs are enriched in tumor-derived genomic material (Amintas et al., 2020). As demonstrated by us and other groups, the DNA can be present as double or single strands and can either be attached to the EV surface or be located inside the EVs protected by the lipid bilayer (Guescini et al., 2010; Cai et al., 2013; Kahlert et al., 2014; Lázaro-Ibáñez et al., 2014; Lázaro-Ibáñez et al., 2019). All of these characteristics make EVs a promising source of cancer biomarkers (San Lucas et al., 2016; Liu et al., 2021).

Most EV studies have focused on cell lines (Crescitelli et al., 2013; Raimondo et al., 2020) or body fluids (San Lucas et al., 2016; Castillo et al., 2018), but these have shown some limitations because the cultured cells may no longer be representative of the tumor because they are influenced by long-term culture and have lost the influence of the tissue microenvironment. Moreover, EVs isolated from body fluids originate from both cancer and non-cancer cells, resulting in a mixture of EVs. To our knowledge, the currently available systems are not sensitive enough to distinguish EVs released by cancer cells from non-cancer-derived EVs. The analysis of EVs directly in the tumor tissue could help to identify EVs released by cancer cells and consequently make the downstream analysis easier. For all these reasons, we recently established a protocol to isolate subpopulations of EVs from metastatic melanoma tissue (Crescitelli et al., 2021).

**FIGURE 1:**
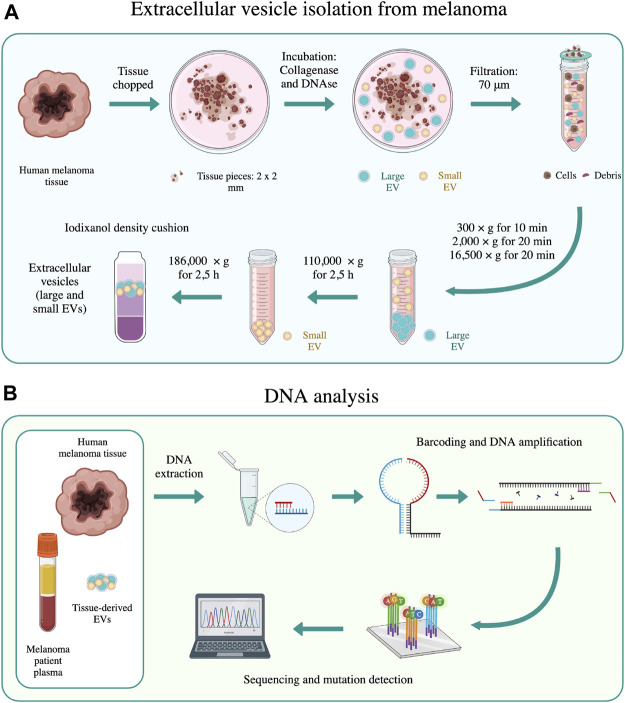
Schematic representation of the main methods used in this work. **(A)** EV isolation from melanoma tissues was performed according to the method of Crescitelli et al. (Crescitelli et al., 2021). **(B)** DNA sequencing was performed on human melanoma tissue, tissue-derived EVs, and melanoma patient plasma using SiMSen-Seq analysis, as described by Ståhlberg et al. (Ståhlberg et al., 2017). The schematic was created with biorender.com.

We and others have previously isolated and analyzed tissue-derived EVs (Perez-Gonzalez et al., 2012; Vella et al., 2017; Cianciaruso et al., 2019; Hurwitz et al., 2019; Huang et al., 2020), but to our knowledge the DNA content has never been described in detail for tumor-specific mutations. The overall aim of the present study was therefore to determine whether EVs present in melanoma tissues contain tumor-derived DNA and to ask whether this could potentially be a more precise source of cancer-specific DNA compared to cfDNA in plasma. To test this hypothesis, we combined the protocol for EV isolation from tissues (Figure 1A) and SiMSen-Seq, a simple multiplexed, PCR-based barcoding of DNA for sensitive mutation detection using sequencing (Figure 1B). The technique involves the use of barcoded primers and error-free sequencing, which enables SiMSen-Seq to bridge the gap between digital PCR and next-generation sequencing (NGS) (Ståhlberg et al., 2017). Digital PCR is a highly sensitive method that allows for a limited number of specific variants to be analyzed, whereas NGS has a broader target capability but is less sensitive in detecting low-frequency mutant alleles.

## 2 Method

### 2.1 Patient information

Metastatic tissue samples and blood samples from six patients with stage III or IV melanoma were collected at the time of surgery, and patient demographics are shown in Supplementary Table S1. Surgery and blood sampling were performed at the Department of Surgery at Sahlgrenska University Hospital from August 2016 to May 2019. Ethical approval was granted by the Regional Ethical Review Board at the University of Gothenburg, Sweden (Dnr #096-12 and 995-16), and the patients provided written consent.

### 2.2 Blood sampling

Peripheral blood was collected in K2E EDTA tubes, and plasma was isolated as previously described (Karimi et al., 2018). Briefly, the blood was centrifuged at 1,880 × *g* for 10 min at room temperature (RT). The plasma was transferred to new tubes and centrifuged at 2,500 × *g* for 10 min at RT, aliquoted, and stored at –80°C until DNA extraction.

### 2.3 Isolation of EVs from human melanoma metastatic tissue

EVs were isolated from melanoma metastases as previously described (Crescitelli et al., 2021) with some minor changes. Briefly, tumor pieces were gently sliced into small fragments (1–2 mm) and incubated with collagenase D (2 mg/ml, Roche, Basel, Switzerland) and DNase I (40 U/ml, Roche) dissolved in plain RPMI medium (Sigma Aldrich, St Louis, MO) for 30 min at 37°C. A separate piece of the tumor that was not used for EV isolation was saved for DNA isolation. After the 30 min incubation, the samples were filtrated through a 70 μm cell strainer. The flowthrough of this filtration step was centrifuged at 300 × *g* for 10 min and 2,000 × *g* for 20 min to further remove cells and tissue debris before the supernatant was stored at –80°C. After thawing, the supernatants were centrifuged at 16,500 × *g*_avg_ (Type 70 Ti (k-factor = 965), Beckman Coulter, Brea, CA) for 20 min and 118,000 × *g*_avg_ (Type 70 Ti (k-factor = 135), Beckman Coulter) for 2.5 h to collect large vesicles and small vesicles, respectively. All centrifugations were performed at 4°C. Pellets were resuspended in PBS. Large and small EVs were combined and further purified by isopycnic centrifugation using an iodixanol gradient (OptiPrep, Sigma-Aldrich, Burlington, MA). Briefly, EVs from tumors tissues in PBS (1 ml) were mixed with 60% iodixanol (3 ml) and laid on the bottom of an ultracentrifuge tube (final volume 4 ml and final concentration 45% iodixanol) followed by the addition of 30% iodixanol (4 ml) and then 10% iodixanol (4 ml). Samples were ultracentrifuged at 97,000 × *g*_avg_ (SW 41 Ti, Beckman Coulter) for 2 h. EVs (∼1 ml) were collected from the interface between the 30% and 10% iodixanol layers. The samples were then mixed with PBS and the EVs were re-pelleted at 118,000 × *g* (Type 70 Ti (k-factor = 266), Beckman Coulter) for 2.5 h and dissolved in PBS.

The schematic overview of the centrifugation-based protocol used to isolate EVs from human melanoma metastatic tissue is shown in Figure 1A.

### 2.4 Transmission electron microscopy

For negative staining, a drop of EVs corresponding to 5 μg of isolated EVs was placed on a 200-mesh formvar/carbon copper grid (glow discharged prior to loading of the sample) (Ted Pella, Redding, CA) for 15 min. The samples were then washed in PBS, fixed in 2% paraformaldehyde for 10 min, further washed in PBS, fixed in 2.5% glutaraldehyde for 10 min, washed in H_2_O, and contrasted in 2% uranyl acetate for 5 min. Images were obtained using a LEO 912AB Omega 120 kV electron microscope (Carl Zeiss SMT, Mainz, Germany). Digital image files were acquired with a Veleta CCD camera (Olympus-SiS, Münster, Germany).

### 2.5 Protein measurement

Protein concentrations of melanoma metastatic tissue-derived EVs were evaluated using Qubit (Thermo Fisher Scientific, San Jose, CA) according to the manufacturer’s protocol.

### 2.6 Western blot

Samples from patients 5 and 6 were loaded and separated on precast 4–20% polyacrylamide Mini-PROTEAN TGX gels (Bio-Rad Laboratories, Hercules, CA). The separation was carried out under reducing conditions for anti-calnexin, anti-flotillin-1, and anti-mitofilin and under non-reducing conditions for anti-CD63, anti-ADAM10, anti-CD9, and anti-CD81 antibodies. After transferring to PVDF membranes (Bio-Rad Laboratories), the membranes were blocked with EveryBlot Blocking Buffer (Bio-Rad Laboratories) for 5 min at RT and then incubated with the following primary antibodies diluted in EveryBlot Blocking Buffer at 4 °C overnight: anti-flotillin-1 (1:1,000 dilution, clone EPR6041, Abcam, Cambridge, United Kingdom), anti-CD9 (1:1,000 dilution, clone MM2/57, Millipore, Darmstadt, Germany), anti-calnexin (1:1,000 dilution, clone C5C9, Cell Signaling Technology, Leiden, Netherlands), anti-CD63 (1:1,000 dilution, clone H5C6, BD Biosciences), anti-CD81 (1:1,000 dilution, clone M38, Abcam), anti-mitofilin (1:500 dilution, polyclonal, Invitrogen, Carlsbad, CA, United States), and anti-ADAM10 (1:500 dilution, clone 163,003, R&D System, Minneapolis, MN, United States). The membranes were washed three times in TBST and then incubated with the appropriate HRP-conjugated secondary antibodies diluted 1:5,000 in EveryBlot Blocking Buffer. The secondary antibodies were sheep anti-mouse IgG HRP-linked F (ab)2 fragment (1:5,000 dilution) and donkey anti-rabbit IgG HRP-linked F (ab)2 fragment (1:5,000 dilution) (both from GE Healthcare, Buckinghamshire, United Kingdom) for 1 h at RT. The membranes were then washed four times for 5 min in TBST and analyzed with the SuperSignal West Femto maximum sensitivity substrate (Thermo Fisher Scientific) on a ChemiDoc Imaging System (Bio-Rad Laboratories).

### 2.7 Single particle interferometric reflectance imaging sensing

EV samples isolated from patient 5 were analyzed with the ExoView™ Plasma Tetraspanin kit and an ExoView™ R100 (NanoView Biosciences, Boston, MA), according to the manufacturer’s instructions as previously described (Crescitelli et al., 2020). The ExoView™ Plasma tetraspanin kit captured the EVs with anti-CD63 (clone H5C6), anti-CD81 (clone JS-81), anti-CD9 (HI9a), and anti-CD41a (clone HIP8), with mouse IgG as the negative control. A total of 50 µl of the sample (1–3*10^8^ particles in total) was mixed with 50 µl of incubation solution, and 35 µl of the diluted samples was added to the chip and incubated at RT for 16 h. The samples were then subjected to immunofluorescence staining using the fluorescent antibodies CD9-CF488 (clone HI9a), CD63-CF647 (clone H5C6), and CD81-CF555 (clone JS-81) that are provided in the ExoView™ Plasma Tetraspanin kit. The samples were washed and then scanned using an ExoView™ R100 imaging system. The data were analyzed using the Nanoviewer analysis software version 2.8.10.

### 2.8 DNA extraction and quantification

#### 2.8.1 DNA extraction from melanoma tissue

The QIAamp DNA mini kit (Qiagen) was used according to the manufacturer’s instructions. Briefly, approximately 25 mg tissue was placed in a tube and 180 µl ATL buffer and 20 µl proteinase K were added. The samples were mixed and incubated at 56°C overnight to dissolve the tissue. The next day the samples were stored at –20°C until DNA was isolated. The samples were thawed, 4 µl RNase A was added, and the samples were mixed and incubated for 2 min at RT. Next, 200 µl AL buffer was added and the samples were mixed and incubated at 70°C for 10 min. Then 200 µl ethanol (96–100%) was added and the samples were mixed. The samples were added to a QIAamp Mini Spin column and centrifuged at 6,000 × *g* for 1 min. The flowthrough was discarded and 500 µl AW1 buffer was added and the samples were centrifuged at 6,000 × *g* for 1 min. The flowthrough was discarded and 500 µl AW2 buffer was added and the samples were centrifuged 20,000 × *g* for 3 min. The QIAamp Mini spin column was placed in a new collection tube and 200 µl AE buffer was added to elute the DNA from the column with a 6,000 × *g* centrifugation for 1 min. Another 100 µl AE buffer was added and the sample was centrifuged again. The two elutions were pooled.

#### 2.8.2 DNA extraction from melanoma tissue-derived EVs

The QIAamp DNA mini kit was used according to the manufacturer’s instructions. Briefly, the samples were thawed and either 400 µl sample was placed in a tube and 40 µl Qiagen Protease, 4 µl RNase A and 400 µl AL buffer were added or 200 µl sample was placed in a tube and 20 µl Qiagen Protease, 2 µl RNase A, and 200 µl AL buffer were added. The samples were mixed and incubated at 56 °C for 10 min, and then 200 µl ethanol (96–100%) was added to all samples irrespective of the starting volume and the samples were mixed. The samples were added to a QIAamp Mini Spin column and centrifuged at 6,000 × *g* for 1 min. The flowthrough was discarded and 500 µl AW1 buffer was added and the samples were centrifuged at 6,000 × *g* for 1 min. The flowthrough was discarded and 500 µl AW2 buffer was added and the samples were centrifuged at 20,000 × *g* for 3 min. The QIAamp Mini spin column was placed in a new collection tube and 50 µl AE buffer was added and the sample was incubated at RT for 5 min. To elute the DNA from the column the samples were centrifuged at 6,000 × *g* for 1 min.

#### 2.8.3 DNA extraction from plasma

The QIAamp Circulating Nucleic Acid Isolation kit (Qiagen) was used according to the manufacturer’s instructions. Briefly, the plasma was thawed and centrifuged at 2,500 × *g* for 10 min, and 3 ml of the supernatant was transferred to a new tube. Next, 300 µl Proteinase K and 2.4 ml ACL buffer containing carrier RNA were added. The samples were vortexed and incubated at 60°C for 30 min. A total of 5.4 ml ACB buffer was added, and the samples were vortexed and put on ice for 5 min. A 20 ml tube extender was placed into an open QIAamp mini column that was positioned in a vacuum system. The samples were added and the vacuum system was turned on. After approximately 10 min the tube extender was removed and 600 µl ACW1 buffer was added to the QIAamp mini column and the vacuum was turned on. The same procedure was performed first with 750 µl ACW2 buffer and then with 750 µl ethanol (96–100%). The QIAamp mini column was removed from the vacuum system and placed in a collection tube and centrifuged at 20,000 × *g* for 3 min. The QIAamp mini column was placed in a new collection tube and incubated with an open lid at 56°C for 10 min to dry the membrane completely. The QIAamp mini column was placed in a new collection tube and 150 µl AVE buffer was added and the sample was incubated at RT for 3 min before the DNA was eluted by centrifuging at 20,000 × *g* for 1 min.

#### 2.8.4 DNA quality controls

DNA fragment length was analyzed using a Bioanalyzer 2,100 instrument with High Sensitivity DNA kits (Agilent Technologies Inc., Palo Alto, CA, United States) according to the manufacturer ´s protocols. DNA concentration was evaluated using the dsDNA High Sensitivity assay (Thermo Fisher Scientific) on a Qubit 2.0 Fluorometer (Invitrogen) according to the manufacturer’s protocol. The concentration of DNA isolated from melanoma tissues, melanoma-derived EVs and plasma samples is shown in Supplementary Table S2.

### 2.9 Library construction and sequencing

SiMSen-Seq was performed for ultrasensitive mutant allele detection as described (Ståhlberg et al., 2017). A multiplex, melanoma-specific panel of 34 assays was used (Supplementary Table S3), and all forward primers included barcodes or unique molecular identifiers (UMIs) that allowed for counting the number of original molecules and provided bioinformatical correction of PCR errors after sequencing. UMIs were protected in a hairpin structure during the first PCR (barcoding step) to prevent non-specific product formation due to off-target binding of the UMIs.

Barcoding of DNA was performed in a reaction containing 0.05 U Platinum™ SuperFi™ DNA Polymerase (Thermo Fisher Scientific), 1× SuperFi Buffer (Thermo Fisher Scientific), 0.2 nM dNTP Mix (Thermo Fisher Scientific), 0.5 M l-carnitine inner salt (Sigma-Aldrich), 40 nM of each barcode primer (Integrated DNA Technologies, Coralville, IA), 4 μl of target DNA, and Ultrapure™ DNase/RNase-Free Distilled Water (Thermo Fisher Scientific) to a total volume of 15 μl. The PCR program was performed in a T100 thermal cycler (Bio-Rad Laboratories) with the following program: 98°C for 30 s, 3 cycles of amplification (98°C for 10 s, 62 °C for 6 min, and 72°C for 30 s with ramping rates of 4°C/s), 65°C for 15 min, and 95°C for 15 min. Before the 15-min incubation at 65°C, 30 μl of 45 ng/μl *Streptomyces griseus* protease (Sigma-Aldrich) dissolved in RNase-free TE buffer (pH 8.0, Thermo Fisher Scientific) was added to each reaction well to reduce non-specific product formation by degrading the DNA polymerase.

Amplification of the previously barcoded product was performed in a second PCR with a total reaction volume of 60 μl containing 1× Q5^®^ Hot Start High-Fidelity Master Mix (New England BioLabs, Ipswich, MA), 400 nM of each Illumina Adapter index primer (desalted, Integrated DNA Technologies, Supplementary Table S3), 15 μl of the barcoded PCR product from previous step, and 10.2 μl of Ultrapure™ DNase/RNase-Free Distilled Water (Thermo Fisher Scientific). The following program was used on a T100 Thermal cycler: 98°C for 3 min, 28 to 30 cycles of amplification (98°C for 10 s, 80°C for 1 s, and 72°C for 30 s with ramping rates of 0.2°C/s).

Libraries were purified with AMPure XP magnetic beads (Beckman Coulter) with a 1:1 volume ratio, and library quality and quantification were determined with the HS NGS Fragment kit (Agilent, Santa Clara, CA) on a Fragment Analyzer using the PROsize software version 3.0 (Agilent). Final quantification of the library pool was performed with NEBNext Library Quant Kit (New England Biolabs) using a CFX384 Touch Real-Time PCR Detection System (Bio-Rad Laboratories). All analyses were performed according to the manufacturer’s instructions.

Sequencing was performed on a MiniSeq (Illumina, San Diego, CA) using a high output reagent kit (150 cycles) with 20% added PhiX control v3 (Illumina) and 1.8 p.m. library. Single read sequencing was performed in 150 cycles.

The schematic overview of the DNA analysis is shown in Figure 1B.

### 2.10 Statistics and bioinformatics

Where appropriate, data are expressed as the mean and standard deviation of the mean (SEM). Statistical analysis was performed by non-paired Student’s t-test or one-way ANOVA for multiple comparisons in GraphPad Prism 6 (GraphPad Software Inc., La Jolla, CA).

The sequencing data were processed using the UMIErrorCorrect software version 0.21. Following the bioinformatics pipeline, reads were mapped, UMIs were extracted, and PCR errors were corrected by forming consensus reads of all sequencing reads with the same UMI because they were all derived from the same original template molecule. We required at least 3 reads per UMI for consensus read generation, subsequently referred to as “consensus 3”. Coverage at consensus 3 was >1,000 for all selected assays in all samples except for the patient 2 plasma sample, which had a coverage of 736. The average coverage at consensus 3 for all samples was 6,059 reads. Consensus output files were analyzed using the UMIAnalyzer R package version 1.0.0 with a consensus depth of 3.

## 3 Results

### 3.1 Isolation and characterization of EVs isolated from human melanoma tissue

**FIGURE 2:**
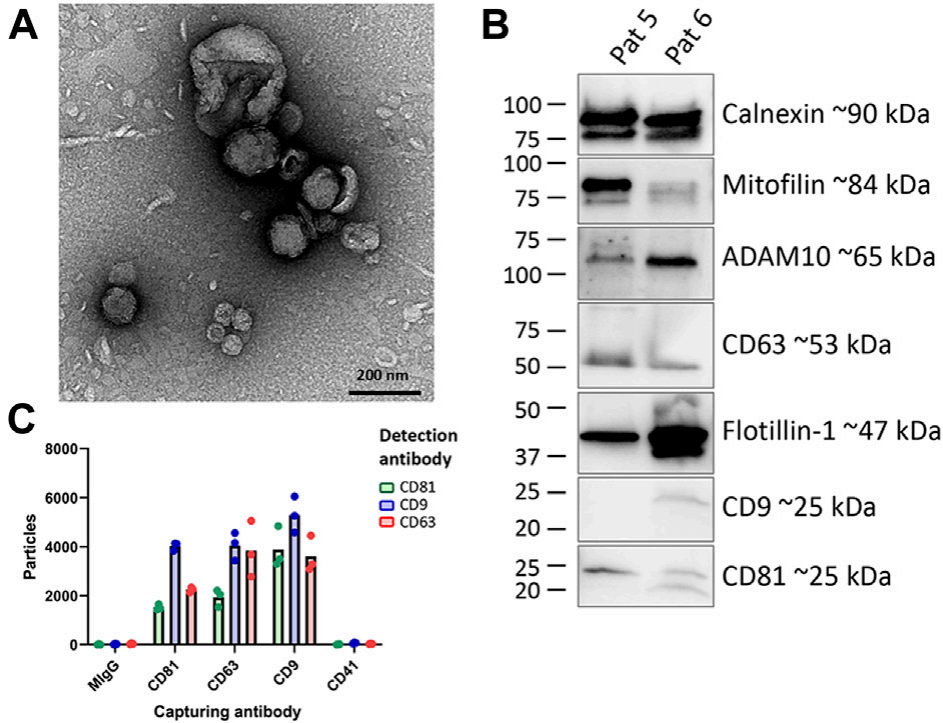
Characterization of EVs isolated from melanoma tissues **(A)** Five micrograms of a mixture of large and small EVs were loaded onto a grid, negative stained, and evaluated with TEM. Scale bars are 200 nm. **(B)** Western blot was used to investigate the presence of the classical vesicle markers flotillin-1, CD63, CD9 and CD81, mitofilin, and ADAM10 as well as the ER protein calnexin in patient 5 and patient 6 **(C)** ExoView™ analysis showing the presence of CD9, CD63, CD81, and CD41a on EVs. The results are presented as the mean, but individual values from three different spots on the chip (technical replicates) are shown (N = 1).

We first characterized the EVs isolated from human melanoma metastatic tissues. TEM images showed the presence of typical large and small EVs (large EVs: 100–300 nm; small EVs: 40–100 nm) (Figure 2A). Furthermore, the background observed on the grids appeared free of non-vesicular contaminants such as large protein structures, indicating that the EV purification was successful (Jang et al., 2019; Crescitelli et al., 2020; Park et al., 2021). The presence of several classical EV markers was determined by Western blot and SP-IRIS (ExoView™). EVs showed positivity for calnexin, a marker of the endoplasmic reticulum, as well as for the classical EV proteins flotillin-1, CD63, CD9, and CD81, which are commonly used to demonstrate the presence of EVs in samples (Théry et al., 2018). Additionally, mitofilin and ADAM10, two recently suggested markers for large and small EVs, respectively, (Kowal et al., 2016; Crescitelli et al., 2020), were also detected in our EV samples (Figure 2B). Moreover, SP-IRIS demonstrated the presence of EVs that were double-positive for CD9, CD63, and CD81 and negative for the platelet marker CD41, which indicated low contamination of blood-derived EVs in the tumor EV isolates (Figure 2C). Together, this shows that a mixture of small and large vesicles positive for commonly used EV-markers had been isolated from the melanoma tissues. These results were in line with our previous findings (Jang et al., 2019; Crescitelli et al., 2020; Park et al., 2021).

### 3.2 Mutant allele frequency analysis in melanoma tissues, melanoma-derived EVs, and plasma

After successful isolation and characterization of melanoma tissue-derived EVs, we focused on analyzing their DNA content compared to DNA isolated directly from tumor tissues, as well as cell-free DNA in plasma. We first performed a DNA qualitative analysis. Bioanalyzer analysis showed that the distribution of DNA fragment sizes in EV was somewhat shorter than the sizes observed in tumor tissue-derived DNA and plasma DNA (Supplementary Figures S1A–C respectively). In comparison, shorter DNA fragments with an average length around 166bp were visible in plasma DNA (Supplementary Figure S1C) (Bronkhorst et al., 2019).

To identify each patient’s tumor-specific mutations, a panel of 34 assays targeting regions of genes known to be frequently mutated in melanoma was used to construct sequencing libraries from the tumor tissue samples using the SiMSen-Seq protocol (Ståhlberg et al., 2017). The assays with the identified mutations were then used to analyze DNA isolated from the tumor-derived EVs and from the plasma samples (Figure 1B). The criteria for the selection of true mutations were mutations identified by routine clinical sequencing and/or mutations with a relatively high allele frequency (>10%), non-synonymous mutations, mutations reported as pathogenic in the literature, and mutations whose assays had no technical issues (such as low coverage or background noise). The mutations that passed these criteria are shown in Table 1. We identified eight mutations in six patients.

**TABLE 1:** Mutations identified in melanoma tissues.

Patient	Mutation status^a^	SiMSen-Seq mutation
1	BRAF wt	CDKN2A p.R80^b^, STK19 p.D89 N
	NRAS wt	
2	BRAF V600K	BRAF p.V600K
	NRAS wt	
3	BRAF V600K	BRAF p.V600K, RAC1 p.P29S
	NRAS wt	
4	BRAF wt	PPP6C p.R264C
	NRAS wt	
5	BRAF V600E	BRAF p.V600E
	NRAS wt	
6	BRAF wt	NRAS p.Q61R
	NRAS Q61R	

^a^ *BRAF*, and *NRAS*, mutational analysis according to routine analysis performed by the Department of Pathology at Sahlgrenska University Hospital.

^b^ Nonsense mutation.

**FIGURE 3:**
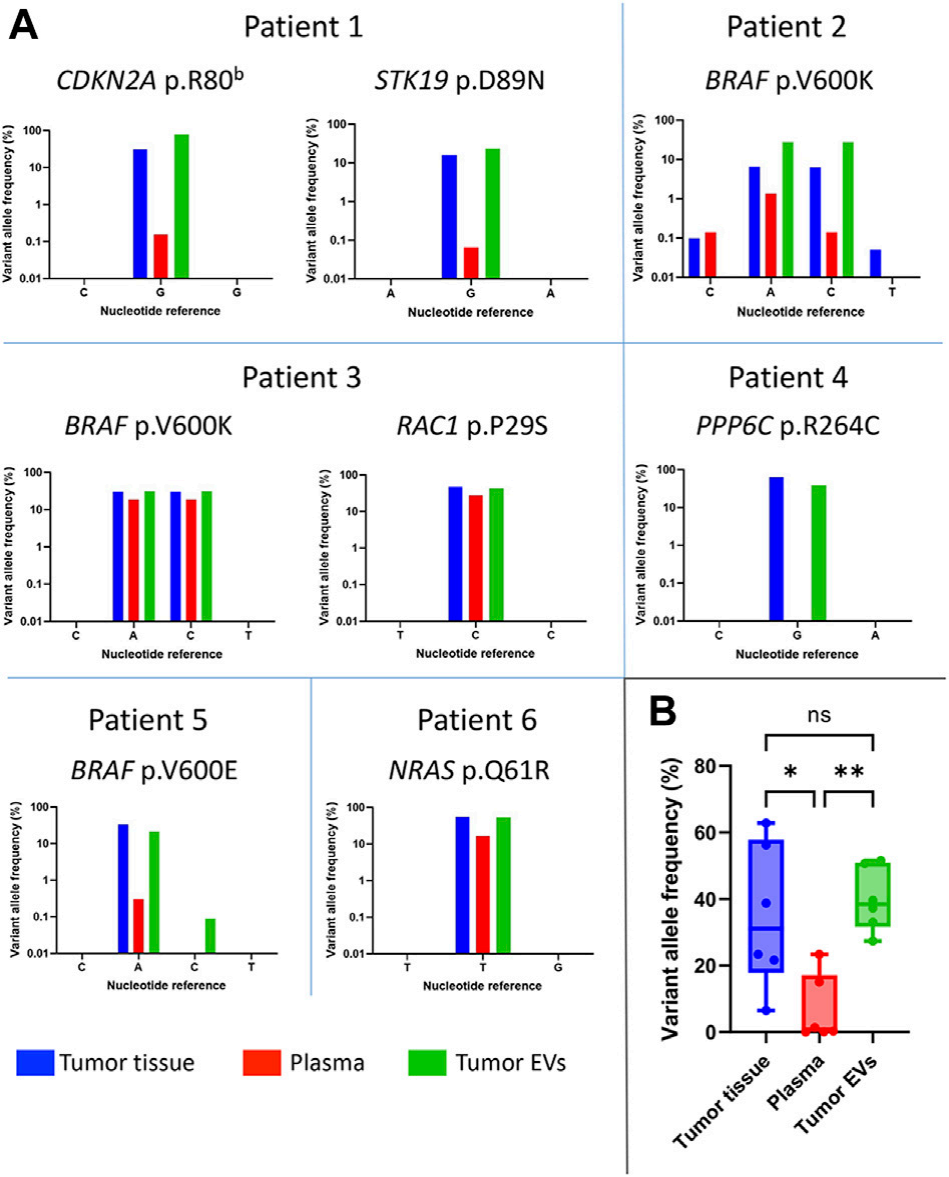
Mutant allele frequencies in melanoma patients. **(A)** Frequencies of the mutated alleles detected in tumor tissue (blue), plasma (red), and tumor-derived EVs (green) are shown per patient **(B)** Visualization of the median frequency of mutated tumor alleles detected in all patients (*n* = 6) per sample type. The allele frequency variations were considered significant with a *p*-value < 0.05 (*p < 0.05; **p < 0.005). Graphs are shown in logarithmic scale.

The mutation status for *BRAF* and *NRAS* in the patient tumors had previously been established by the Department of Pathology at Sahlgrenska University Hospital, and the results obtained for the melanoma tissues with the SiMSen-Seq technique regarding these two specific genes were in accordance with the previously established mutation (Table 1). *BRAF* mutations were found in patients 2, 3, and 5, all carrying V600 E/K mutations, while a *NRAS* ^*Q61R*^ mutation was detected only in patient 6. Additionally, the SiMSen-Seq technique identified a *CDKN2A* and a *STK1*9 mutation in patient 1, a *RAC1* mutation in patient 3, and a *PPP6C* mutation in patient 4. The patient-specific assays containing these mutations (Table 1) were used to determine whether these mutations could also be detected in tissue-derived EVs and in plasma DNA. All mutations detected in the tumor tissue could also be detected in the tissue-derived EVs (Figure 3A). The mutant allele frequencies of tumor-derived EVs were higher or similar to those in the tumor samples. It is noteworthy that the mutant allele frequencies from tumor samples showed a wider dispersion, ranging from 6.5% to 62.8% (median = 31.1%), while samples from tumor-derived EVs ranged from 23.7% to 51.6% (median = 38.4%). Interestingly, the mutant allele frequencies from the plasma samples were consistently lower for every mutation, ranging from below the limit of detection to 27.9%, with a median value of 0.8% (Figure 3B). Together, these results show that the mutations identified in melanoma tissue could also be detected in tissue-derived EVs at a high allele frequency.

## 4 Discussion

In this study we combined two innovative techniques–the isolation of EVs from melanoma tumor tissues and the ultrasensitive SiMSen-Seq method–to determine the presence of DNA mutations in tissue-derived EVs. The SiMSen-Seq method detected a high frequency of mutations in tumor-derived EVs, indicating that a significant portion of the EVs originated from the malignant cells. We identified a total of six mutations in the EV DNA, including mutations in the *BRAF*, *NRAS*, *CDKN2A*, *STK19*, *PPP6C*, and *RAC* genes in different patients. *BRAF* mutations were present in three patients and one patient had an *NRAS* mutation, whereas two patients had wild type *BRAF* along with *NRAS* mutations in the EV DNA. The mutations observed in the EVs were identical to those identified by routine clinical analysis.

The variant allele frequencies were higher in tissue-derived EVs compared to plasma DNA, although with varying levels. Of note, in one patient, the *BRAF* mutation allele frequency observed in tissue-derived EVs was higher than in the tissue. The reasons are not clear but a lower allele frequency in tissue could be explained by differences in tumor heterogeneity and a higher stromal content. In tissue-derived EVs, the allele frequency could instead be enriched due to cancer cells secreting more EVs than e.g. stromal cells, thereby increasing the allele frequency (Vasconcelos et al., 2019).

We have previously been able to isolate EVs from both metastatic melanoma tissues as well as other tumors (Crescitelli et al., 2021). Tissue-derived EVs have previously been shown to carry RNA and traditional EV proteins (Vella et al., 2017; Hurwitz et al., 2019; Huang et al., 2020), but to our knowledge this is the first study to describe DNA in tissue-derived EVs. Importantly, our current tissue EV isolation protocol includes the use of DNase, which should remove the DNA that we know can be present on the EV surface (Lázaro-Ibáñez et al., 2019), but not the DNA inside of the EVs (Cai et al., 2013; Kahlert et al., 2014; Lázaro-Ibáñez et al., 2019) because the enzyme does not penetrate membranes, and we therefore suggest that the mutation-rich DNA in EVs is likely present inside their membranes.

Even though cfDNA is considered a promising tool for mutation analysis in cancer, and even though SiMSen-Seq is one of the most sensitive techniques for identifying such mutations, we were repeatedly unable to identify mutations in the plasma, even though the patients had obvious mutations. However, the mutated cfDNA represents only a small fraction of the total free DNA in the circulation, making the identification of low-frequency tumor mutations challenging. This technique is, however, much more sensitive than NGS, which analyses the DNA broadly, but it is less effective at identifying rare mutations (Fox et al., 2014). Moreover, SiMSen-Seq overcomes the limit of digital PCR, which only allows a limited number of variants to be analyzed. Here we have been able to analyze, in a single assay, 34 different DNA mutations relevant to melanoma. For melanoma, *BRAF* and *NRAS* mutations are considered to be important disease-driving oncogenes and were included in the mutation analysis performed in this study. We found mutations in *BRAF* and *NRAS* using SiMSen-Seq, thus confirming the results obtained from routine clinical analysis. However, we complemented the analysis with other mutations not investigated in the routine analysis, including *CDKN2A*, *STK19*, *PPP6C*, and *RAC*. Even though SiMSen-Seq is sensitive, it seems from our data that it can yield false negative results in the plasma of metastatic patients. However, we were able to clarify that the DNA mutations occur at high frequency in tumor EVs. Therefore, capturing tumor-specific EVs from the circulation could potentially increase the sensitivity of the cfDNA analysis using SiMSen-Seq, and we suggest that efforts to develop such techniques could be helpful for the field.

Isolating EVs from any source, including cell culture medium, biofluids, or tissues, is not trivial, and the methods need to be adapted depending on the source of EVs and the scientific questions being asked. In the case presented here, isolating EVs from tissues may to some degree capture vesicles that have an intracellular origin. Although the dissection of tissues was performed with the utmost care, some cells are likely to have been disrupted, and their cytosolic material may have been co-isolated with the EVs. However, we are confident that the EV fractions indeed are at least enriched in EVs because TEM, western blots, and SP-IRIS analyses all confirmed the presence of EV markers in the isolated tissue EVs.

In this work we have successfully combined two innovative techniques–the isolation of EVs from tissues and NGS using the SiMSen-Seq assay–thus demonstrating that DNA is present in EVs isolated from tissues and that the DNA contains the same mutations found in tissue samples from the same patient. Although further studies are needed to validate these findings in larger cohorts of patients, this work paves the way for the use of EV tumor-derived DNA in melanoma diagnosis. In the future, the technique may be even more clinically applicable if methods for the specific capture of tumor EVs in circulation can be developed.

## Data Availability

The original contributions presented in the study are included in the article/Supplementary Materials, further inquiries can be directed to the corresponding authors.
