# Supplementary figures and images for: Extracellular vesicle DNA from human melanoma tissues contains cancer-specific mutations
Source: Front Cell Dev Biol. 2022 Dec 1;10:1028854. doi: 10.3389/fcell.2022.1028854 (PMC9751452; doi:10.3389/fcell.2022.1028854)

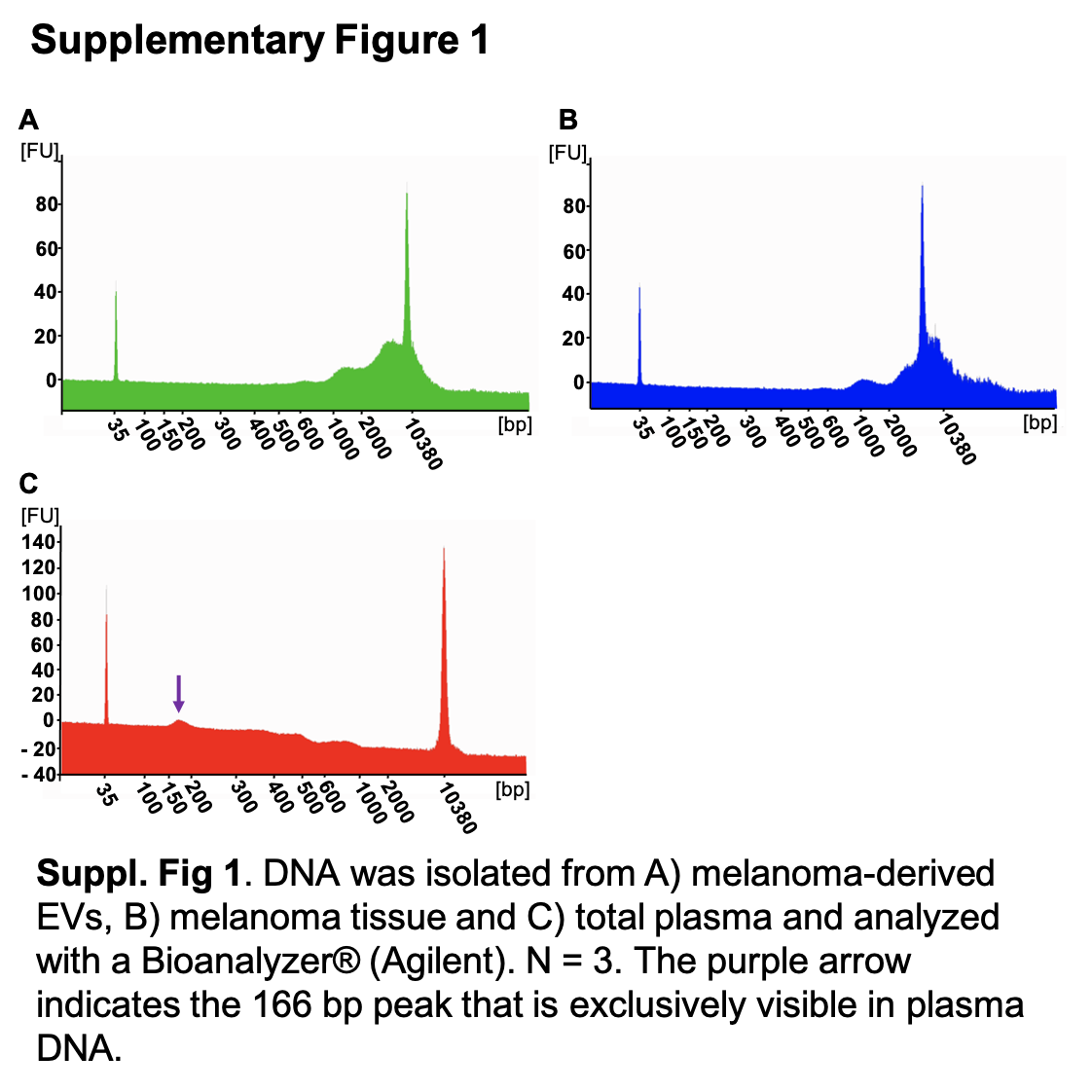

Supplement: Supplementary file 3 [file Image1.tif]
